# RITA requires eIF2α-dependent modulation of mRNA translation for its anti-cancer activity

**DOI:** 10.1038/s41419-019-2074-3

**Published:** 2019-11-07

**Authors:** Johannes Ristau, Vincent van Hoef, Sylvain Peuget, Jiawei Zhu, Bo-Jhih Guan, Shuo Liang, Maria Hatzoglou, Ivan Topisirovic, Galina Selivanova, Ola Larsson

**Affiliations:** 1 Department of Onkology-Pathology, Science for Life Laboratories, Karolinska Institutet, Stockholm, Sweden; 2 Department of Microbiology, Tumor and Cell Biology, Karolinska Institutet, Stockholm, Sweden; 3 Department of Genetics and Genome Sciences, Case Western Reserve University, Cleveland, OH USA; 4 Lady Davis Institute, SMBD JGH and Gerald Bronfman Department of Oncology, McGill University, Montreal, QC Canada

**Keywords:** Drug development, Apoptosis

## Abstract

Tumor protein 53 (p53, encoded by the *TP53* gene) is a key tumor suppressor regulating cell fates in response to internal and external stresses. As *TP53* is mutated or silenced in a majority of tumors, reactivation of p53 by small molecules represents a promising strategy in cancer therapeutics. One such agent is RITA (reactivation of p53 and induction of tumor cell apoptosis), which restores p53 expression in cells with hyperactive HDM2 and induces apoptosis. Yet, mechanisms underlying the anticancer activity of RITA are incompletely understood. Here we show that RITA suppresses mRNA translation independently of p53 by inducing eIF2α phosphorylation. Surprisingly, reactivation of p53 following RITA treatment is critically dependent on eIF2α phosphorylation. Moreover, inhibition of eIF2α phosphorylation attenuates pro-apoptotic and anti-neoplastic effects of RITA, while inducing phosphorylation of eIF2α enhances the anticancer activity of RITA. Collectively, these findings demonstrate that the translational machinery plays a major role in determining the antineoplastic activity of RITA, and suggest that combining p53 activators and translation modulators may be beneficial.

## Introduction

Multiple stress signals, including DNA damage and oncogenic signaling, converge to increase the level and activity of p53 which in turn orchestrates gene expression programs to determine cell fate decisions and suppress neoplastic transitions^1,2^. It is thus generally thought that cells restrict p53 activity to progress towards malignancy^3,4^. Consistently, a pan-cancer study identified *TP53* as the single most mutated gene^5^. Moreover, in tumors with wild-type *TP53*, expression of the p53 protein is commonly repressed via overexpression of HDM2 or HDMX which target p53 for proteasomal degradation^6,7^. Reactivation of p53 therefore holds significant therapeutic promise, but it is necessary to consider the variety of mechanisms which inactivate normal p53 function in neoplasia when devising such approaches^8^. To this end, a range of small molecules have been identified including those refolding mutant p53 (e.g. PRIMA-1^9^) or inhibiting proteasome dependent degradation of wild-type p53 (e.g., Reactivation of p53 and Induction of Tumor Apoptosis [RITA]^10^, Nutlin-3a^11^, SAR405838^12^). Although clinical trials using p53 activators have been conducted^13^ or are still ongoing (NCT01143519, NCT01636479), the downstream mechanisms which mediate the effects of these drugs on, e.g., apoptosis and/or cell cycle upon p53 reactivation are incompletely understood. Perplexingly, these agents do sometimes not solely rely on restoring p53 transactivation^14–16^ and, in some cases, anticancer effects are observed independently of TP53^17^. This suggests that reactivation of p53 function acts in concert with other mechanisms and/or that reactivation of p53 may be a secondary effect of drug action. Indeed, several mechanisms have been proposed to explain *TP53*-independent induction of cell death following treatment with p53 reactivating drugs including DNA damage^18^, endoplasmic reticulum (ER) stress^19^ and an imbalance in glutathione (GSH) vs. reactive oxygen species (ROS) production^15^. These studies suggest that characterization of downstream mechanisms of p53 reactivation agents may allow for rational selection of patients for treatment and/or improve design of drug combinations encompassing p53 activators.

Multiple cellular stresses activate the integrated stress response (ISR) to globally suppress protein synthesis (reviewed in ref. ^20^). This is chiefly achieved via the phosphorylation^21^ of the alpha subunit of the eukaryotic translation initiation factor 2 (eIF2α) which delivers initiator tRNA during translation initiation in a ternary complex (TC) with GTP^21^. Upon initiator tRNA delivery, eIF2-GTP is hydrolyzed to GDP and subsequently recycled by eIF2B which acts as a guanine nucleotide exchange factor (GEF) for the next round of initiation^21^. eIF2α phosphorylation (reviewed in ref. ^22^), by one of four stress sensing kinases (PKR-like endoplasmic reticulum kinase [PERK], Heme-regulated inhibitor [HRI], general control nonderepressible-2 [GCN2] or Protein Kinase RNA-activated [PKR]^23–26^) inhibits the GEF activity of eIF2B thereby preventing TC recycling. This leads to suppression of global protein synthesis, which co-occurs with translational activation of a subset of mRNAs, with specific features in their 5′ un-translated regions (UTR) including inhibitory upstream open reading frames (uORFs), which encode stress-induced factors including activating transcription factor 4 (ATF4) and C/EBP homologous protein (CHOP). If the stress is resolved, eIF2α is dephosphorylated by the GADD34:PP1 complex^27^, and protein synthesis recovers (reviewed in ref. ^22^). This recovery also involves translational reprogramming that allows translation of mRNAs that support stress resolution^28^. Unresolved stress, however, often results in cell death which is mediated by factors such as CHOP and binding immunoglobulin protein (BIP)^29^.

RITA was discovered in a small molecule screen designed to identify compounds restoring wild-type p53 activity and is thought to inhibit the interaction between p53 and HDM2^10^. Further studies suggested that RITA induces apoptosis independent of p53^16,17,30^. Here we show that RITA induces apoptosis and represses mRNA translation by inducing eIF2α phosphorylation independent of p53; and, surprisingly, that reactivation of p53 following RITA treatment requires eIF2α phosphorylation. Moreover, modulation of eIF2α phosphorylation largely accounts for the antineoplastic effects of RITA in cell culture. Thus, modulation of mRNA translation appears to be required for the anti-tumor effects of RITA.

## Materials and methods

### Cell lines and reagents

MCF7 (WT, *TP53+/+*, *TP53−/*−, sh-control, *sh4EBP1*, MCF7 *TP53−/− shD133/D160p53*), GP5D and HT1080 cells were maintained in Dulbecco’s Modified Eagle Medium (Gibco Thermo Fisher, Gothenburg, Sweden) with 1% Penicillin-Streptomycin (Thermo Fisher), 1% GlutaMAX (Thermo Fisher) and 10% Fetal Bovine Serum (Thermo Fisher). HCT116 *TP53+/+* cells were maintained in McCoy’s 5 A (Modified) Medium (Thermo Fisher) with 1% Penicillin-Streptomycin and 10% Fetal Bovine Serum. MCF7 *TP53+/+* and MCF7 *TP53−/−* cells were generated by CRISPR/Cas9 mediated *TP53* deletion (TGAAGCTCCCAGAATGCCAG) as described^31^. Briefly, stable Cas9 expressing MCF7 were established and then transfected two times with *TP53* sgRNA targeting exon 4. Cell lines were obtained as follows: MCF7 WT cells were purchased from Sigma Aldrich (Schnelldorf, Germany). MCF7 *sh4EBP1*, HT1080 WT, HT1080 S51A were received from Ivan Topisirovic; HCT116 *TP53+/+* and GP5d cells were received from Galina Selivanova. Cells were cultured to a maximum of 15 passages (<2 months) after thawing and all experiments where performed during this period. Mycoplasma testing was performed by PCR [primers: GGCGAATGGGTGAGTAACACG (forward) and CGGATAACGCTTGCGACTATG (reversed); samples were compared to a positive and negative control] after at least 2 days after thawing and monthly. RITA (2443/1) and GSK2606414 (5107) were purchased from Tocris (Bristol, United Kingdom). Integrated stress response inhibitor (ISRIB; SML0843), N-actetyl cystein NAC (A9165) and salubrinal (SML0951) were purchased from Sigma-Aldrich.

### Polysome-profiling

Cells were seeded in 15 cm culture dishes and harvested at ~75% confluence. Following treatment, cytosolic and polysome-associated RNA were extracted as described previously^32^. After sedimentation of the cytosolic lysate in the sucrose gradient, absorbance at 254 nm was recorded along the gradient, resulting in polysome-tracings. Overlays of tracings were normalized for input material and quantification was performed by measuring the area under the curve for efficiently translated mRNA (herein defined as association with >3 ribosomes).

### ^[35]^S-methionine/cysteine labeling

^[35]^S-labeled methionine and ^[35]^S-labeled cysteine incorporation in nascent proteins was measured according to the manufacturer’s instruction (EasyTag EXPRESS^35^S Protein Labeling Mix, Perkin Elmer, Upplands Väsby, Sweden). Briefly, 10^5^ cells were seeded per well in six well plates, allowed to attach overnight and treated in methionine and cysteine free DMEM (Gibco Thermo Fisher) with RITA in presence or absence of ISRIB at indicated concentrations for 4 h. Next, cells were incubated for 30 min in DMEM supplemented with S35 labeled Met and Cys (20 µCi/ml), after which they were washed three times with PBS and lysed with 100 µl radio-immunoprecipitation assay buffer (RIPA buffer; 100 mM sodium chloride, 1.0% Triton X-100, 0.5% sodium deoxycholate, 0.1% SDS, 50 mM Tris pH 8.0 [Sigma-Aldrich]). The lysate was centrifuged for 10 min at 20.000 rpm in a tabletop centrifuge and 15 µl of the supernatant was spotted on a glass fiber filtermat (Filtermat B, Perkin-Elmer). The filtermat was subsequently washed twice in 10% Trichloroacetic acid (TCA) and once with ethanol:acetone (50:50) for 10 min each and dried overnight. A melt-on scintillator (MeltiLex, Perkin-Elmer) was applied to the filtermat and counts per minute were monitored using a microBeta plate reader (MicroBeta2, Perkin Elmer).

### ROS detection using CellROX

Endogenous ROS levels were detected using the CellROX Deep Red Reagent (C10422, ThermoFisher Scientific). MCF7 cells were grown to 70% confluency prior to 16 h incubation with 5 µM N-acetyl cysteine with or without 1 µM RITA added during the last 4 h. After this, the Deep Red reagent was added to the culture medium for 30 min at a final concentration of 5 µM after which the cells were washed three times with PBS and analyzed by FACS. Normalization to the control condition and plotting was done using FCS Express 6 Plus Research Edition (DE Novo Software, Glendale, CA, USA).

### ROS detection using DCFDA

One million cells were seeded in 6 cm dishes. The day after, cells were treated as indicated, then washed with PBS and incubated 30 min with 10 μM DCFDA (ThermoFisher Scientific) in serum free medium. Cells were then trypsinized, washed twice with PBS and fluorescence was analyzed by a FACSCalibur flow cytometer (BD Biosciences, Stockholm, Sweden) using CellQuest Pro software (BD Biosciences).

### Western blotting

Whole cell lysates were extracted using RIPA buffer supplemented with phosphatase and protease inhibitors (Roche PhosSTOP and cOmplete tablets). 20 µg of protein was subjected to SDS-PAGE using 10% or 13% Bis-Acrylamide gels (29:1) (Sigma-Aldrich) before transfer to a 0.2 µm nitrocellulose membrane (BioRad, Solna, Sweden). All antibodies were used in 4% Bovin serum albumin dissolved in TBS-buffer (20 mM Tris, 150 mM NaCl) and 0.1% Tween 20. Primary antibodies used in this study, were incubated under constant agitation at 4 °C for 16 h: P53, DO-1, Santa Cruz Biotechnology (Heidelberg, Germany), 1:800; beta-actin, Sigma-Aldrich, 1:10,000; PARP (46D11), Cell Signaling Technologies 9532 S, 1:1000; phospho-4EBP1 (S65), Cell Signaling Technologies (purchased via BioNordika Sweden, Stockholm, Sweden), 9456 S, 1:1000; 4EBP1, Cell Signaling Technologies, 9452S, 1:1000; phoshpo-S6K (Thr389), Cell Signaling Technologies, 9234S, 1:1000; total S6K, Cell Signaling Technologies, 9202S, 1:1000; phospho-eIF2α (S51), Cell Signaling Technologies, 9721S, 1:1000; eIF2α, Cell Signaling Technologies, 9722S, 1:1000.

Secondary antibodies used in this study were incubated under constant agitation at RT for 30 min: goat anti-rabbit, BioRad, 19205 S, 1:10,000; goat anti-mouse, Merck Millipore (Schnelldorf, Germany), AP127P, 1:10000. To re-probe membranes with additional antibodies, stripping was performed using Restore Western Blot Stripping Buffer (Thermo Fisher Scientific) for 15 min at RT and subsequent blocked using 4% BSA in TBS-T for 30 min. Before exposure, and between incubations of primary and secondary antibodies, membranes were washed three times with TBS-T. Proteins were visualized using Clarity Western ECL Substrate (BioRad) on a Thermo Fisher iBright CL1000 system.

### Lentiviral transduction of the D133/D160p53 shRNA construct

HEK293T cells were transfected with pCMV-VSVG, pMDLg-RRE, pRSV-REV and a transfer vector with an shRNA (5′-GACTCCAGTGGTAATCTAC-3′) targeting exon 7 of *TP53* (D133/D160p53 shRNA) using a Calcium Phosphate Transfection Kit (CAPH05-1KT, Sigma Aldrich). The supernatant was collected 48 h after transfection and filtered (0.45 µm). MCF7 *TP53−/−* were then transduced using 8 µg/mL polybrene and media was changed after 16 h. This generated MCF-7 *TP53−/− shD133/D160p53* cells.

### Reverse transcription and qPCR

400.000 MCF-7 *TP53−/− shD133/D160p53* cells were seeded per well in a 6-well plate and treated with 1 μM RITA the following day. RNA was extracted using Aurum total RNA mini Kit (7326820, BioRad). 1 µg of total RNA was used for reverse transcription using iscript cDNA synthesis kit (1708891, BioRad) according to manufacturer’s instructions. qPCR was performed using the Premix Ex Taq Kit (RR390W, Takara, Gothenburg, Sweden) using the following protocol: 95 °C, 30 s for one cycle followed by 40 cycles of 95 °C, 5 s; 60 °C, 30 s. The following primers were used: Beta actin: fwd. 5′TTCTACAATGAGCTGCGTGTG3′, rev. 5′GGGTGTTGAAGGTCTCAAA amplification efficiency 2.05; D133/D160p53 fwd: GTGCAGCTGTGGGTTGATTC, rev: ACCATCGCTATCTGAGCAGC, amplification efficiency 2.11. Ct values were normalized to actin and expression was calculated using the ΔΔCt-method.

### Flow cytometry and Annexin V-propidium iodide staining

Adherent and floating cells were collected by centrifugation (1200 × *g* for 5 min in a tabletop centrifuge) and stained using the Dead Cell Apoptosis with FITC AnnexinV/PI kit according to manufacturer’s instructions (Life technologies Thermo Fisher, V13242). Fluoresence was analyzed using an ACEA NovoCyte flow cytometer (ACEAbio, AH diagnostics, Aarhus, Denmark), and further normalized to cell counts using FCS Express 6.

### Colony formation assay

Colony formation was assessed by seeding 1000 MCF7 cells per well in a 6-well plate (Corning). After 16 h, cells were treated with the indicated compounds for 4 h. Media was changed every three days. After 10 days, colonies were stained with Crystal Violet staining solution [0.4% Crystal violet (Sigma-Aldrich), 6% Glutaraldehyde (Sigma-Aldrich) in H2O] for 30 min on an orbital shaker (20 rpm) and de-stained with ddH2O. Quantification was performed using OpenCFU (http://opencfu.sourceforge.net/) with the following parameters: Threshold: regular = 5, Radius = 5, Auto = max. Data was normalized to a DMSO control and the experiment was performed three times where each experiment included a technical duplicate.

### Statistical analysis

Experiments were performed at least three times (*n* = 3) unless otherwise stated in the figure legend. All replicates were biological. Statistics were calculated using two-sided unpaired Student’s *t*-tests in which equal variance was assumed (R studio Version 3.4.3) using a minimum of three biological replicates unless otherwise indicated.

## Results

### RITA induces PARP cleavage and represses mRNA translation in a *TP53-*independent manner

**Fig. 1:**
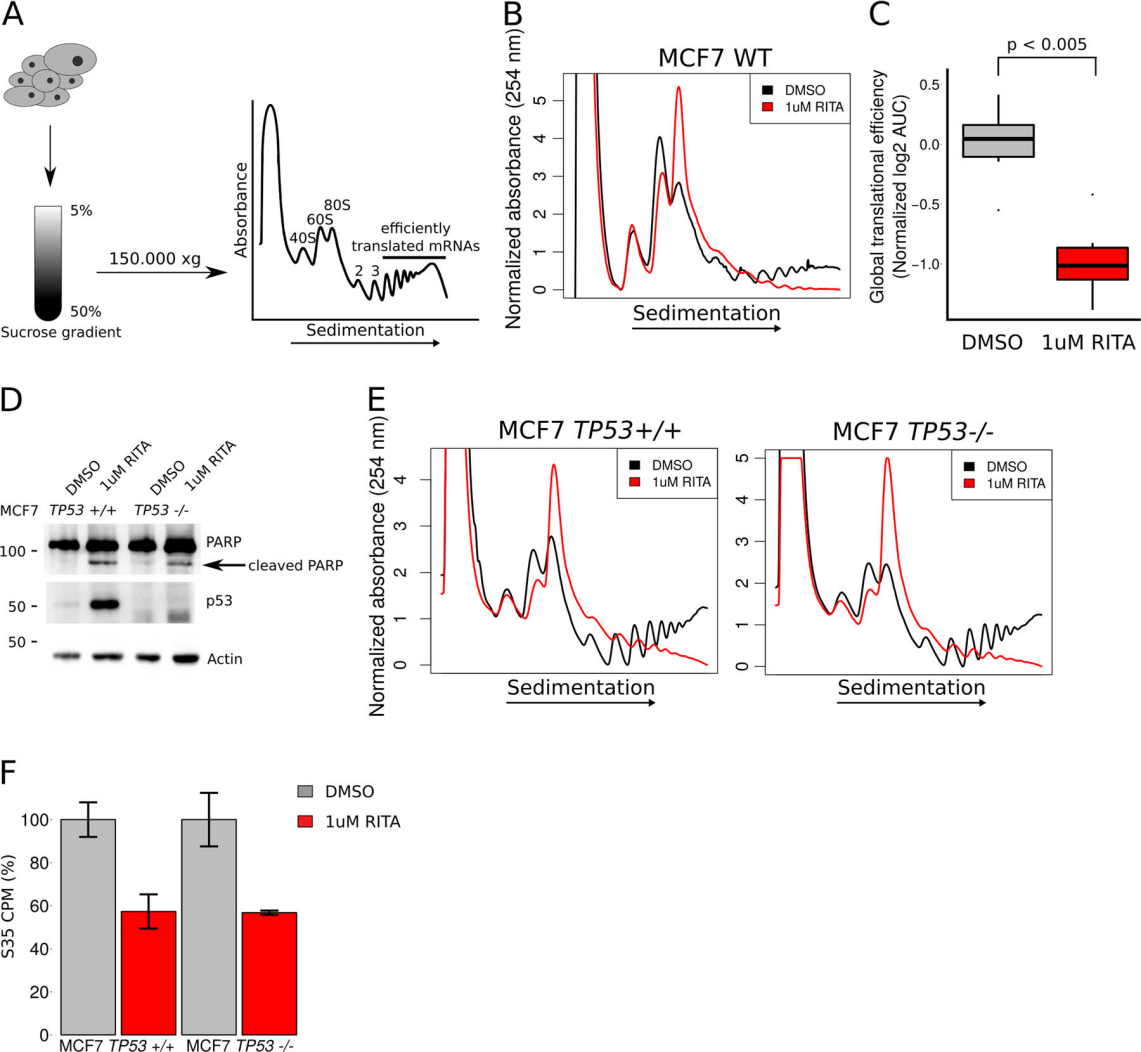
RITA induces PARP cleavage and represses translation in a *TP53-* independent manner. **a** A schematic overview of the polysome-profiling procedure. During polysome-profiling, cytosolic cell lysates are sedimented on a sucrose gradient and relative amount of ribosomes engaged in efficient translation (i.e., >3 ribosomes on an mRNA) can be quantified by calculating the area under the curve (AUC). **b** Representative polysome-tracings of MCF7 WT cells treated with vehicle (DMSO) or 1 µM RITA for 8 h. **c** Quantification of changes in amounts of efficiently translated mRNA using polysome-tracings from MCF7 WT cells treated with vehicle (DMSO) or 1 µM RITA for 8 h (normalized to mean of vehicle treated cells; *n* = 6). **d** Western blotting of indicated proteins in extracts from MCF7 *TP53+/+* and *TP53−/−* cells treated with vehicle (DMSO) or 1 µM RITA for 8 h. **e** Representative polysome-tracings of MCF7 *TP53+/+* and *TP53−/−* cells treated with vehicle (DMSO) or 1 µM RITA for 8 h. **f** Quantification of ^35^S labeled nascent proteins in MCF7 *TP53+/+* and *TP53−/−* cells treated with vehicle (DMSO) or 1 µM RITA for 8 h (normalized to mean of vehicle treated MCF7 *TP53+/+* or *TP53−/−* cells; *n* = 3, bars represent the mean +/− SD)

As previously described, 8 h treatment of MCF7 cells with 1 µM RITA results in a strong p53 accumulation and cleavage of the apoptosis marker PARP (Fig. S1). Perturbations in translation have been linked to both changes in p53 activity and cell death^33,34^. We therefore explored the effects of RITA on protein synthesis using the polysome profiling technique, which separates mRNAs according to the number of associated ribosomes^32^ (Fig. 1a). This allows for visualization and quantification of ribosomes engaged in efficient translation (herein defined as those mRNAs associated with >3 ribosomes) which reflects global translation levels (Fig. 1a). Polysome-profiling analysis revealed that RITA reduces the number of ribosomes engaged in efficient translation with a concomitant increase in 80S monosomes (Fig. 1b, c). Although the effects of RITA were firstly attributed to p53 activation, a recent study revealed that RITA may exert p53-independent effects^16,17^. To assess whether effects of RITA on mRNA translation require p53 we employed MCF7 cells wherein *TP53* was silenced using CRIPSR-Cas9 technology. Strikingly, RITA had a similar effect on PARP cleavage (Fig. 1d) and global translation levels, assessed by polysome profiling, in MCF7 *TP53−/−* as compared to MCF7 *TP53+/+* cells (Fig. 1e). To further quantify RITA’s effects on global translational in the context of p53, we measured incorporation of ^[35]^S-methionine and ^[35]^S-cysteine in nascent proteins following RITA treatment. This confirmed a comparable reduction of protein synthesis in *TP53+/+* and *TP53−/−* MCF7 cells upon RITA treatment (Fig. 1f). Thus, RITA induces PARP cleavage and suppresses global translational efficiency independent of *TP53*.

### RITA suppresses translation independently of ROS and the mTOR/4E-BP axis

**Fig. 2:**
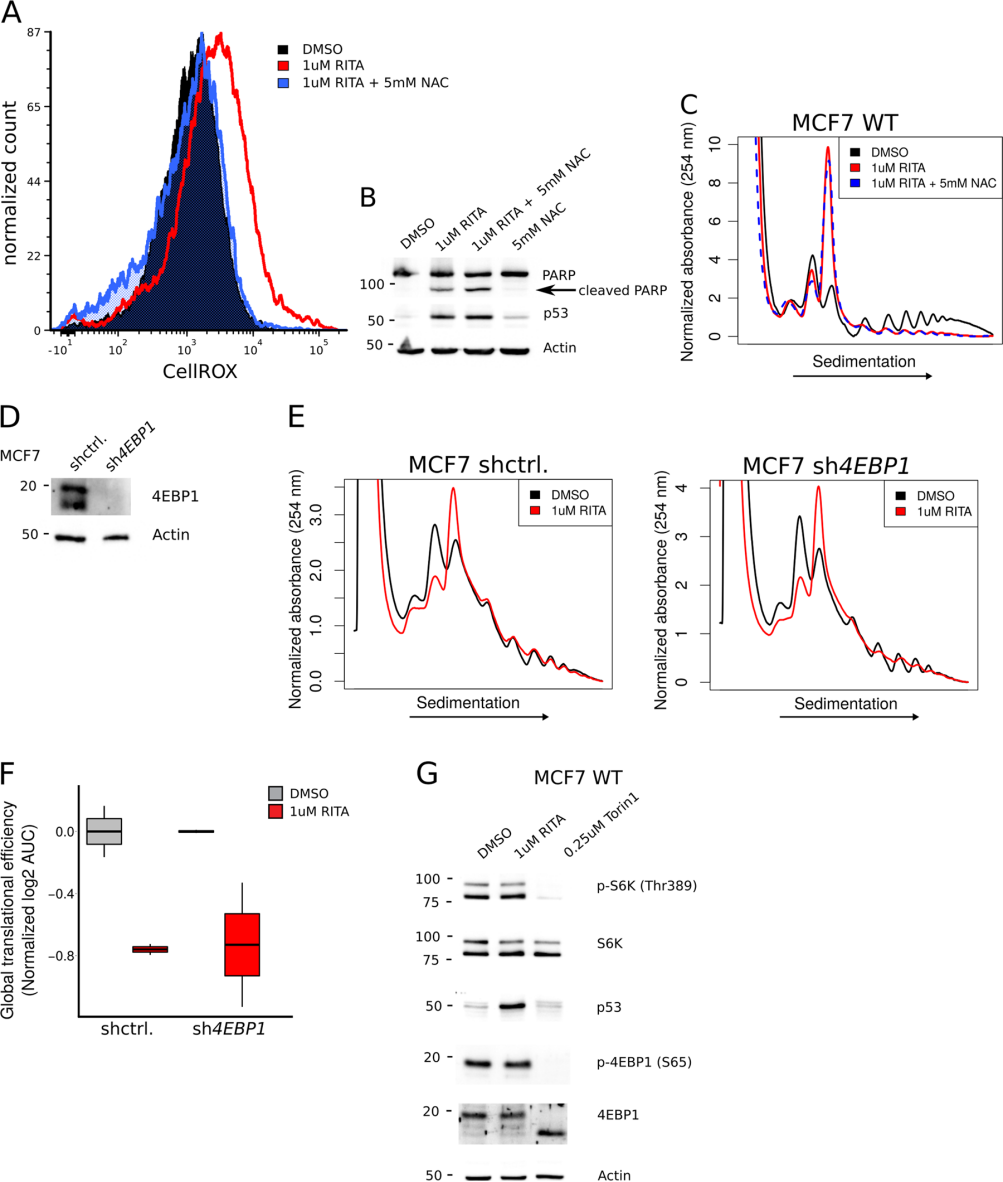
Suppression of translation following RITA treatment does not depend on ROS accumulation or inhibition of the mTOR/4E-BP axis. **a** FACS for CellROX Deep Red dye (to quantify ROS) following an 8 h treatment of MCF7 WT cells with vehicle (DMSO) or 1 µm RITA in presence or absence of 5 mM N-Acetyl Cysteine (NAC). **b** Western blotting for indicated proteins using extracts from MCF7 WT cells treated with vehicle (DMSO) or 1 µm RITA in presence or absence of 5 mM NAC. **c** Polysome-tracings of MCF7 WT cells treated with vehicle (DMSO) or 1 µM RITA in presence or absence of 5 mM NAC for 8 h. **d** Western blotting of indicated proteins using extracts from sh-control and *sh4EBP1* MCF7 cells. **e** Representative polysome tracings of sh-control. and *sh4EBP1* MCF7 cells treated with vehicle (DMSO) or 1 µM RITA for 8 h. **f** Quantification of amount of efficiently translated mRNA from polysome-tracings normalized to the mean of vehicle (DMSO) treated sh-control MCF7 cells (*n* = 2). **g** Western blot for indicated proteins using extracts from MCF7 WT cells treated with vehicle (DMSO), 1 µM RITA or 0.25 µM Torin1 for 8 h

The pro-apoptotic effects of RITA were previously reported to be potentiated by accumulation of ROS^35^. Moreover, recent reports indicate that other p53 reactivating drugs such as PRIMA-1Met may exert *TP53*-independent effects on apoptosis by altering GSH/ROS production^15^. Because oxidative stress can suppress protein synthesis^36^, we assessed whether RITA-induced oxidative stress explains its suppressive effect on mRNA translation. As described previously^35^, 8 h treatment of MCF7 cells with 1 µM RITA resulted in an elevation of ROS as measured by staining with CellROX Deep Red dye (Fig. 2a). Although RITA-induced ROS accumulation could be completely reversed using the anti-oxidant N-Acetyl Cysteine (NAC; Fig. 2a), PARP cleavage persisted (Fig. 2b), p53 levels were elevated (Fig. 2b) and mRNA translation was decreased (Fig. 2c). Thus, RITA-associated accumulation of ROS does not explain its effects on mRNA translation.

The mTOR pathway is a major regulator of protein synthesis and integrates multiple internal and external cues such as nutrient, oxygen, energy and growth factor availability^37^. A key link between mTOR and mRNA translation is mediated by its phosphorylation and inactivation of the small translation-inhibitory 4E-binding proteins (4E-BPs). 4E-BPs bind to the 5′ cap-binding protein eIF4E and prevent assembly of the eIF4F complex^22,38^. Therefore, to investigate the role of the mTOR/4E-BP axis in mediating the effects of RITA on mRNA translation, we used MCF7 cells where 4E-BP1 expression was silenced using a short hairpin RNA (sh4EBP1; Fig. 2d) and assessed the effect of RITA (1 µM for 8 h) on mRNA translation. As assessed by polysome-tracings, cells lacking 4E-BP1 showed a similar reduction in the proportion of ribosomes engaged in efficient translation as compared to control cells (Fig. 2e, f). Moreover, in contrast to treatment with the active site mTOR inhibitor torin1^39^, following RITA treatment we observed no change in phosphorylation of 4E-BP1 or S6K which are downstream targets of mTOR (Fig. 2g). Thus, RITA modulates translation independently of the mTOR/4E-BP axis.

### RITA suppresses translation by inducing phosphorylation of eIF2α

**Fig. 3:**
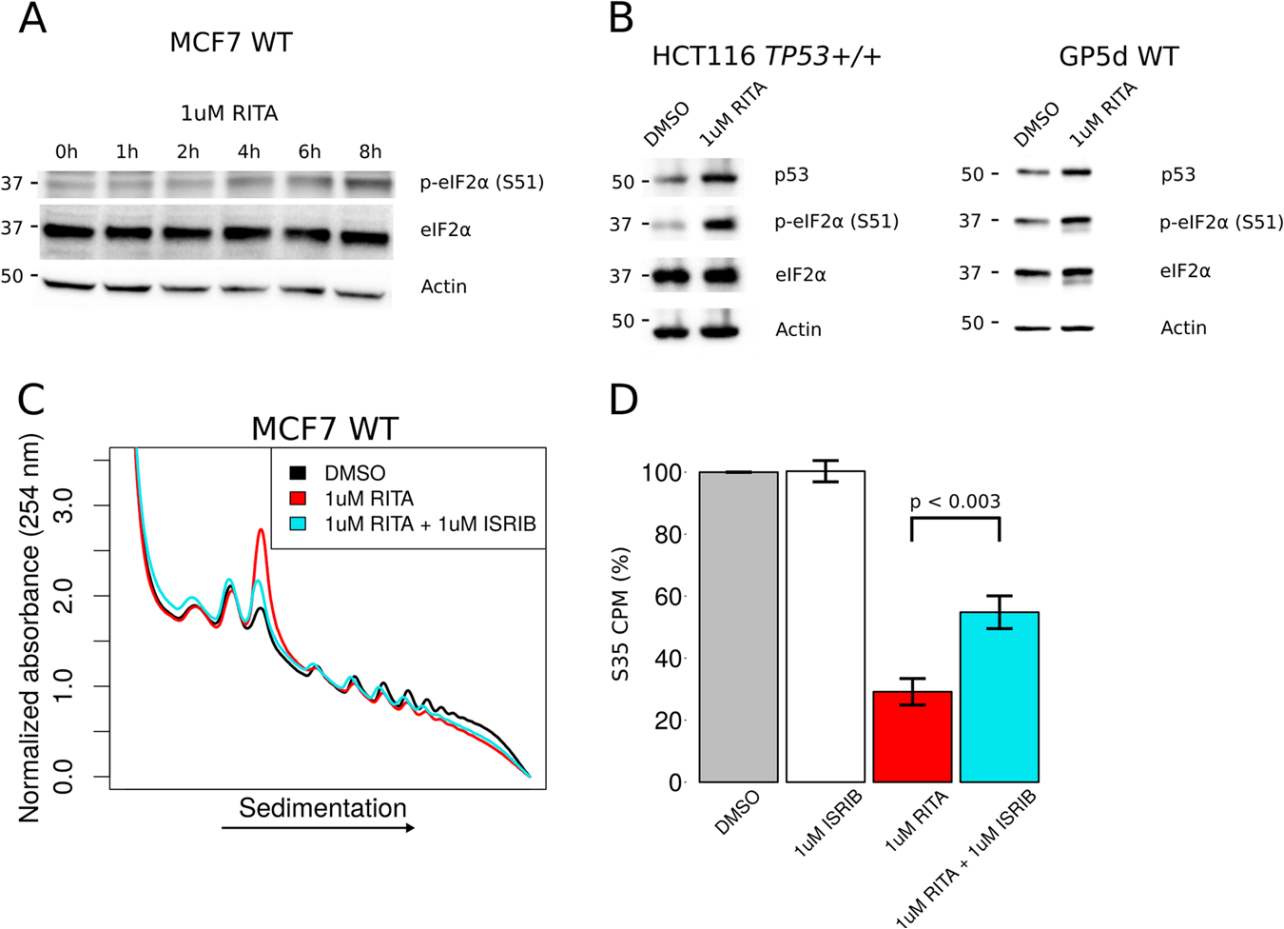
RITA suppresses translation by inducing phosphorylation of eIF2α. **a** Western blot analysis for indicated proteins using extracts from MCF7 WT cells treated with 1 µM RITA for 1–8 h. **b** Western blot analysis for indicated proteins using extracts from HCT116 *TP53+/+* or GP5d WT cells treated with vehicle (DMSO) or 1 µM RITA for 4 h. **c** Polysome profiles of MCF7 WT cells treated with vehicle (DMSO) or 1 µM RITA in presence or absence of 1 µM ISRIB for 4 h. **d** Quantification of S35 labeled nascent peptides from MCF7 WT cells following treatment with vehicle (DMSO) or 1 µM RITA in presence or absence of 1 µM ISRIB for 4 h. Counts per minute (CPM) were normalized to the mean of the vehicle treated cells (*n* = 3, bars represent the mean +/− SD)

Another key step modulating translation initiation is recycling of the TC. Therefore, we next assessed whether the effects of RITA on mRNA translation involve changes in phosphorylation of eIF2α. Indeed, 1 µM RITA treatment was associated with a time-dependent increase in phosphorylation of eIF2α in MCF7 WT cells (Fig. 3a). The effect of RITA on eIF2α phosphorylation was not limited to MCF7 cells as phosphorylation of eIF2α was also induced in the colon cancer cell lines HCT116 *TP53+/+* and GP5d, which express wild-type p53 (Fig. 3b). In addition to full length p53, whose exon 4 was targeted by CRISPR/Cas9 in MCF7 *TP53−/−* cells, *TP53* is also transcribed as a shorter isoform (encoding for both D133p53 and D160p53 protein isoforms; Fig. S2A) which does not include exon 4^40^. As these shorter isoforms of p53 are implicated in tumorigenesis and response to DNA damage^41,42^, we assessed whether effects from RITA on eIF2α phosphorylation and PARP cleavage depend on D133p53 and D160p53. To this end we transduced MCF7 *TP53−/−* cells with an shRNA targeting exon 7, which resulted in an approximate fourfold reduction in the mRNA encoding D133p53 and D160p53 as measured by qPCR (MCF7 *TP53−/− shD133/D160p53* cells*;* Fig S2A, B). Indeed, PARP was cleaved and eIF2α phosphorylated following RITA treatment in MCF7 *TP53−/− shD133/D160p53* cells, which is consistent with RITA-associated anti-cancer effects also independent of D133/D160p53.

To evaluate whether phosphorylation of eIF2α is necessary for suppression of translation by RITA, we employed the integrated stress response inhibitor (ISRIB)^43^. ISRIB induces eIF2B GEF activity and TC recycling in spite of eIF2α being phosphorylated^44^. Indeed, ISRIB partially restored translation following RITA treatment as judged by a decrease in the monosome (80S) peak in polysome-tracings (Fig. 3c) and a concomitant increase in ^[35]^S-methionine incorporation into nascent peptides (Fig. 3d). Thus, the effects of RITA on translation appear to be mediated by eIF2α phosphorylation.

### RITA-mediated suppression of mRNA translation requires PERK activity

**Fig. 4:**
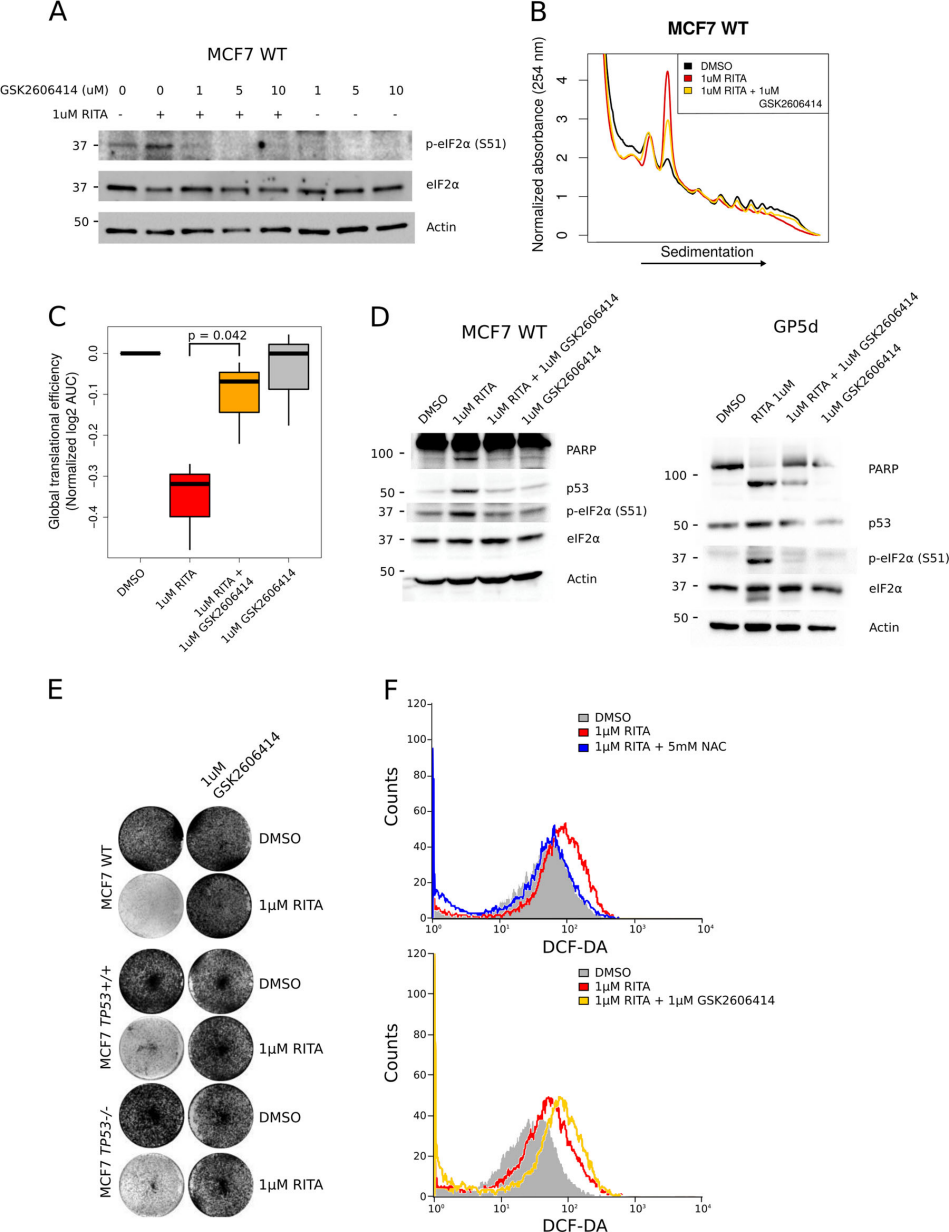
RITA-mediated suppression of mRNA translation requires PERK activity. **a** Western Blot analysis using extracts from MCF7 WT cells treated with vehicle (DMSO) or 1 µm RITA under increasing concentrations of GSK2606414 for 4 h. **b** Representative polysome-tracings of MCF7 WT cells treated with vehicle (DMSO) or 1 µM RITA in presence of absence of 1 µM GSK2606414 for 4 h. **c** Quantification of amount of efficiently translated mRNA from polysome-tracings normalized to the mean of vehicle treated MCF7 WT cells. (*n* = 3, paired *t*-test between RITA and RITA + GSK2606414) **d**) Western blotting using extracts from MCF7 WT and GP5d cells treated with vehicle (DMSO) or 1 µM RITA in presence or absence of 1 µM GSK2606414. **e** Crystal violet staining of MCF7 WT, *TP53+/+* and *TP53−/−* cells treated with vehicle (DMSO) or 1 µM RITA in presence or absence of 1 µM GSK2606414 for 8 h. **f** FACS for DCF-DA (to quantify ROS) in MCF7 WT cells treated with vehicle (DMSO) or 1 µM RITA in presence or absence of 5 mM NAC (upper) or 1 µM GSK2606414 (lower) for 8 h

As discussed above, eIF2α can be phosphorylated by four kinases, each activated by distinct cellular stresses (reviewed in ref. ^45^). Among these, PERK is activated via phosphorylation upon ER stress^23^ and multiple reports have indicated a potential role for ER stress during apoptosis-induction following reactivation of p53^46,47^. This highlighted the possibility that suppression of protein synthesis following RITA treatment may depend on PERK. Indeed, PERK inhibition using GSK2606414^48^ reduced RITA-induced eIF2α phosphorylation (Fig. 4a). This associated with a partial rescue of translation in RITA-treated cells, as judged by a re-distribution of ribosomes from monosomes to polysomes (Fig. 4b, c), together with a reduction in PARP cleavage in MCF7 and GP5d cells (Fig. 4d). Moreover, under PERK inhibition, p53 was no longer induced by RITA (Fig. 4d). Nevertheless, consistent with *TP53*-independent effects on mRNA translation and PARP cleavage (Fig. 1b–d, Fig. S2), cell viability was restored in a *TP53*-independent manner when RITA was used in combination with the PERK inhibitor GSK2606414 (Fig. 4e). Moreover, consistent with ROS independent effects of RITA on mRNA translation and PARP cleavage (Fig. 2b, c), GSK2606414 increased rather than reduced levels of ROS following RITA treatment (Fig. 4f).

### eIF2α phosphorylation status determines the efficacy of RITA

**Fig. 5:**
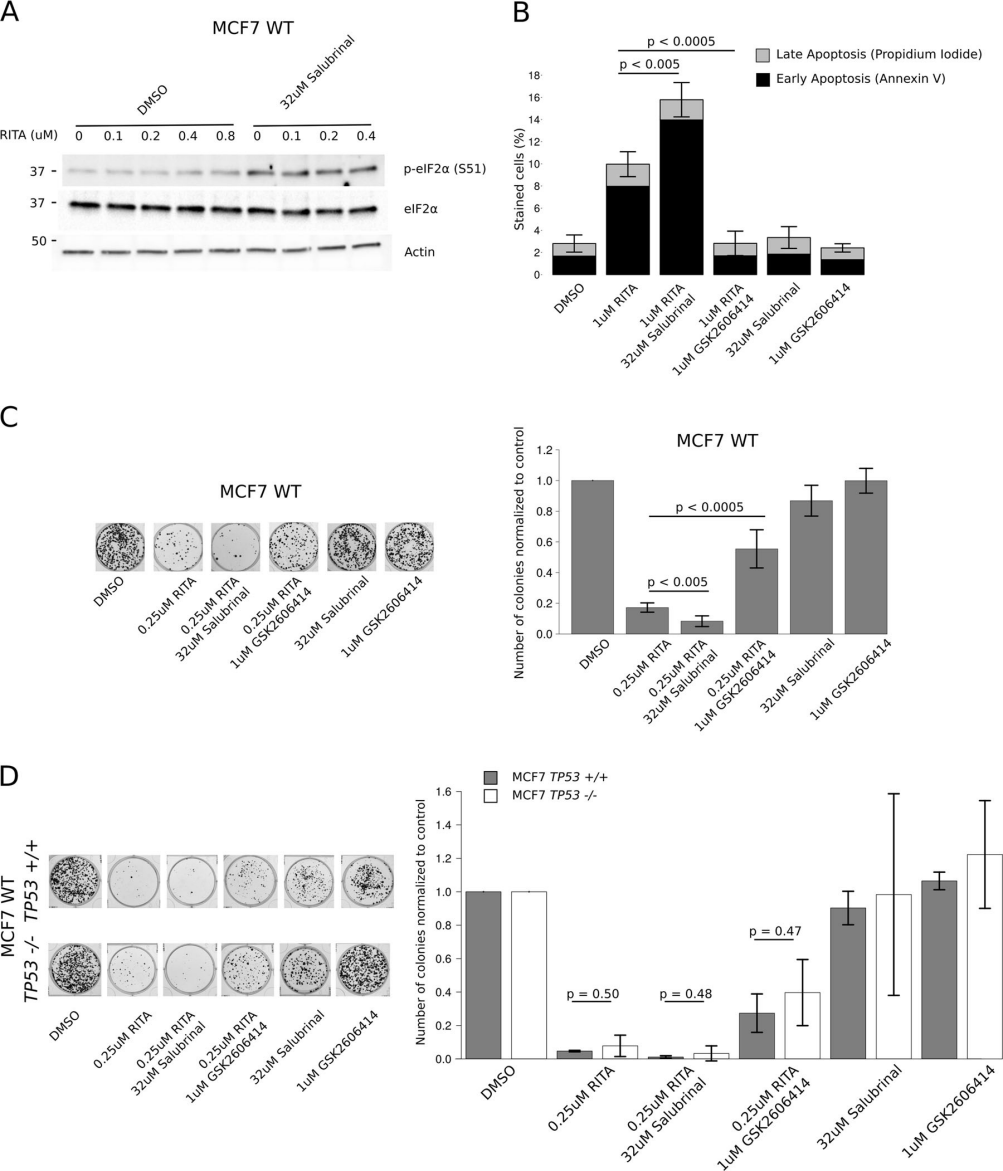
Phosphorylation of eIF2α modulates RITA’s effect on apoptosis and colony formation. **a** Western blot analysis using extracts from MCF7 WT cells treated with increasing concentrations of RITA in presence or absence of 32 µM salubrinal for 4 h. **b** FACS based quantification of Annexin V and propidium iodide staining to detect early and late apoptosis in MCF7 WT cells treated with vehicle (DMSO) or 1 µM RITA in presence or absence of 32 µM salubrinal or 1 µM GSK2606414 for 4 h (*n* = 3). **c**, **d** Crystal violet staining of WT (**c**), *TP53+/+* or *TP53−/−* MCF7 cells (**d**) after treatment with vehicle (DMSO) or 1 µM RITA in presence or absence of 32 µM salubrinal or 1 µM GSK2606414 (4 h treatment followed by a 10 day expansion before staining). The pictures represent representative images and quantification was performed on *n* = 3 experiments. All bars represent the mean +/− SD

These results suggest that eIF2α phosphorylation and subsequent suppression of mRNA translation are correlated with the pro-apoptotic effects of RITA. To explore the causality of this relationship, we suppressed or stimulated eIF2α phosphorylation using the PERK inhibitor GSK2606414^48^ or salubrinal, which inhibits eIF2α phosphatases and thereby enhances phosphorylation of eIF2α [ref. ^49^, Fig. 5a], respectively. GSK2606414 (1 µM) reversed induction of apoptosis induced by RITA (1 µM) (Fig. 5b) as assessed by Annexin V/propidium iodide (PI) staining. Conversely, treatment with 32 µM salubrinal in combination with 1 µM RITA potentiated induction of apoptosis as compared to RITA alone (Fig. 5b). Moreover, salubrinal (32 µM) potentiated inhibitory effects of RITA (1 µM) on colony formation in MCF7 cells, while GSK26064141 (1 µM) caused the opposite effect (Fig. 5c). Consistent with a *TP53*-independent effect on mRNA translation and PARP cleavage (Fig. 1d–f), *TP53* status did not exert a major effect on colony formation in MCF7 cells across the treatments (Fig. 5d).

**Fig. 6:**
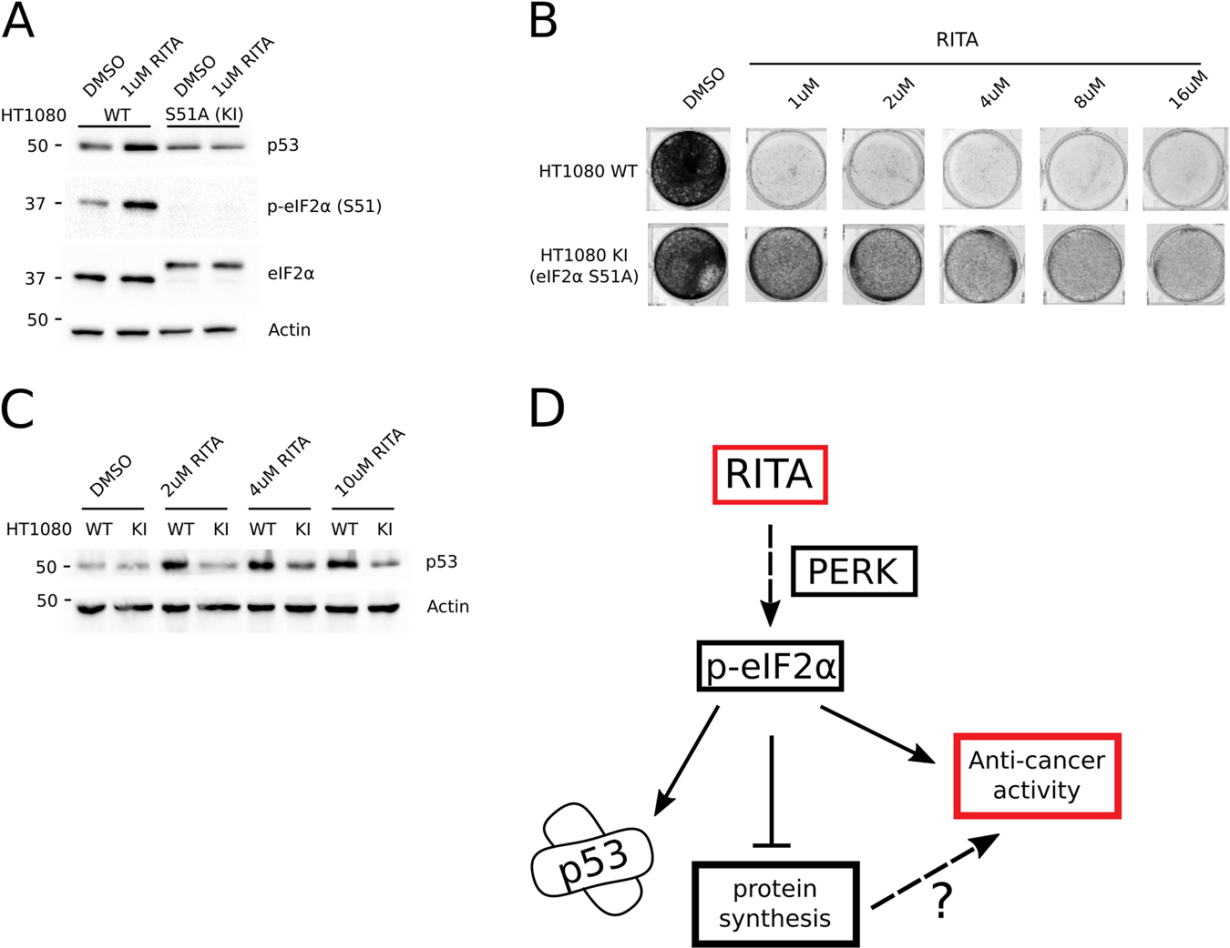
RITA’s effects on clonogenicity and p53 activation depend on phosphorylation of eIF2alpha. **a** Western blot analysis of indicated proteins using extracts from HT1080 WT and HT1080 KI (eIF2αS51A) cells treated with vehicle (DMSO) or 1 µM RITA for 8 h. **b** Crystal violet staining of HT1080 WT and HT1080 KI cells treated with increasing concentrations of RITA. **c** Western blot analysis of indicated proteins using extracts from HT1080 WT and KI cells treated with vehicle (DMSO) or increasing concentrations of RITA for 8 h. **d** Schematic of RITA’s p-eIF2α dependent effects on p53 reactivation, protein synthesis and anti-cancer activity

To further establish the relationship between the antineoplastic effects of RITA and eIF2α phosphorylation, we employed the wild-type *TP53* lung fibrosarcoma cell line HT1080 expressing either wild-type (HT1080 WT) or a non-phosphorylatable eIF2α mutant (serine 51 to alanine mutation; HT1080 KI)^50^ (Fig. 6a). Strikingly, the inability of HT1080 KI cells to phosphorylate eIF2α was associated with increased cell viability under a range of RITA concentrations (1–16 µM) (Fig. 6b). Moreover, RITA-induced p53 in HT1080 WT, but not KI cells (Fig. 6c). This is consistent with the attenuation of p53 induction by RITA under conditions wherein eIF2α phosphorylation is reduced by PERK inhibition (Fig. 4d). Collectively, these findings support a model whereby induction of p53 by RITA depends on phosphorylation of eIF2α (Fig. 6d).

## Discussion

RITA has been demonstrated to prevent proteasomal degradation of p53 by inhibiting the p53-MDM2 interaction^51^. However, as shown here and previously in HCT116 cells^17^, RITA appears to exert its antineoplastic effects independently of *TP53*. Interestingly, several p53 reactivating drugs elicit effects that are independent or only partially dependent on the presence of p53. For example PRIMA-1Met, which reactivates mutant p53, currently in phase I/II clinical trials, has been shown to reduce phosphorylation of MEK, independently of *TP53*, and thereby impair anchorage-independent growth^14^. Moreover, PRIMA-1Met disrupts the GSH/ROS balance and induces autophagy and apoptosis irrespective of *TP53* status^15,52^. Similarly, in multiple myeloma, PRIMA-1Met induces ER stress through p73 demethylation and cells without *TP53* showed the highest drug sensitivity^19^. Finally, p53 was shown to be dispensable for a decrease in clonogenic potential of several cell lines following RITA treatment, which instead correlated with the induction of DNA damage^53^. Strikingly, many of these *TP53*-independent effects impinge on the eIF2α-dependent regulation of mRNA translation [ref. ^36^ and reviewed in ref. ^54^]. Consistently, RITA dramatically induced eIF2α phosphorylation, whereby phospho-eIF2α appears to be a critical mediator of the proapoptotic and antineoplastic effects of RITA independently of p53 including its isoforms D133p53 and D160p53. This is in accordance with a recent study showing an increased radio-sensitivity of cervical cancer cell lines in the presence of RITA, which required induction of ER-stress but occurred independently of *TP53*^55^.

Preventing phosphorylation of eIF2α via PERK inhibition alleviated the pro-apoptotic effects of RITA and partially restored the ability of unlimited cell-division independently of *TP53*. This supports the tenet that PERK, although essentially pro-survival, can also promote apoptotic cell death in a context dependent fashion^56^. Moreover, it has been shown that the persistent activation of PERK in the absence of IRE1 and ATF6 activity during prolonged ER stress represents a terminal pro-apoptotic stage of the unfolded protein response^57^. Accordingly, augmenting the induction of eIF2α phosphorylation by RITA using salubrinal resulted in more potent pro-apoptotic and antineoplastic effects relative to using RITA alone. A similar effect may account for the improved activity of RITA in increasing radio-sensitivity of cervical cancer cell lines treated with RITA^55^.

Overall, we provide evidence that RITA selectively induces PERK activity thereby effectively suppressing mRNA translation and inducing apoptosis in a *TP53*-independent manner. Moreover, these results point to the importance of the stress sensing eIF2α pathway in deciding the cellular fate in the context of RITA, and possibly other p53 reactivating agents, and show that modulation of this pathway may be exploited for therapeutic purposes.

## Supplementary information

Supplemental Figure 1

Supplemental Figure 2

Supplemental Figure Legends no marked changes

Author contribution file

## References

[1] Horn H, Vousden K (2007). Coping with stress: multiple ways to activate p53. Oncogene.

[2] Lazo PA (2017). Reverting p53 activation after recovery of cellular stress to resume with cell cycle progression. Cell. Signal..

[3] Bieging KT, Mello SS, Attardi LD (2014). Unravelling mechanisms of p53-mediated tumour suppression. Nat. Rev. Cancer.

[4] Joerger AC, Fersht AR (2016). The p53 pathway: origins, inactivation in cancer, and emerging therapeutic approaches. Annu. Rev. Biochem..

[5] Kandoth C (2013). Mutational landscape and significance across 12 major cancer types. Nature.

[6] Momand J, Zambetti GP, Olson DC, George D, Levine’ AJ (1992). The mdm-2 oncogene product forms a complex with the p53 protein and inhibits p53-mediated transactivation. Cell.

[7] Linares LK, Hengstermann A, Ciechanover A, Müller S, Scheffner M (2003). HdmX stimulates Hdm2-mediated ubiquitination and degradation of p53. Proc. Natl Acad. Sci. USA.

[8] Zawacka-Pankau J, Selivanova G (2015). Pharmacological reactivation of p53 as a strategy to treat cancer. J. Intern. Med..

[9] Bykov VJN (2002). Restoration of the tumor suppressor function to mutant p53 by a low-molecular-weight compound. Nat. Med..

[10] Issaeva N (2004). Small molecule RITA binds to p53, blocks p53-HDM-2 interaction and activates p53 function in tumors. Nat. Med..

[11] Vassilev LT (2004). In vivo activation of the p53 pathway by small-molecule antagonists of MDM2. Science.

[12] Wang S (2014). SAR405838: an optimized inhibitor of MDM2-p53 interaction that induces complete and durable tumor regression. Cancer Res..

[13] Lehmann S (2012). Targeting p53 in vivo: a first-in-human study with p53-targeting compound APR-246 in refractory hematologic malignancies and prostate cancer. J. Clin. Oncol..

[14] Lu T (2016). PRIMA-1Met suppresses colorectal cancer independent of p53 by targeting MEK. Oncotarget.

[15] Tessoulin B (2014). PRIMA-1Met induces myeloma cell death independent of p53 by impairing the GSH/ROS balance. Blood.

[16] Weilbacher A, Gutekunst M, Oren M, Aulitzky WE, van der Kuip H (2014). RITA can induce cell death in p53-defective cells independently of p53 function via activation of JNK/SAPK and p38. Cell Death Dis..

[17] Wanzel M (2016). CRISPR-Cas9-based target validation for p53-reactivating model compounds. Nat. Chem. Biol..

[18] Valentine JM, Kumar S, Moumen A (2011). A p53-independent role for the MDM2 antagonist Nutlin-3 in DNA damage response initiation. BMC Cancer.

[19] Teoh PJ (2016). PRIMA-1 targets the vulnerability of multiple myeloma of deregulated protein homeostasis through the perturbation of ER stress via p73 demethylation. Oncotarget.

[20] Wek RC, Jiang H-Y, Anthony TG (2006). Coping with stress: eIF2 kinases and translational control. Biochemical Soc. Trans..

[21] Hinnebusch AG (2014). The scanning mechanism of eukaryotic translation initiation. Annu. Rev. Biochem..

[22] Sonenberg N, Hinnebusch AG (2009). Regulation of translation initiation in eukaryotes: mechanisms and biological targets. Cell.

[23] Harding HP, Zhang Y, Ron D (1999). Protein translation and folding are coupled by an endoplasmic-reticulum-resident kinase. Nature.

[24] Vattem, K. M., Staschke, K. A. & Wek, R. C. Mechanism of activation of the double-stranded-RNA-dependent protein kinase, PKR Role of dimerization and cellular localization in the stimulation of PKR phosphorylation of eukaryotic initiation factor-2 (eIF2). *Eur. J. Biochem*. **268**, 3674–3684 (2001). 10.1046/j.1432-1327.2001.02273.x 11432733

[25] Chen J-J, London IM (1995). Regulation of protein synthesis by heme-regulated eIF-2α kinase. Trends Biochemical Sci..

[26] Hinnebusch AG (2005). Translational regulation of *GCN4* and the general amino acid control of yeast. Annu. Rev. Microbiol..

[27] Choy MS (2015). Structural and functional analysis of the GADD34:PP1 eIF2α phosphatase. Cell Rep..

[28] Guan B-J (2017). A unique ISR program determines cellular responses to chronic stress. Mol. Cell.

[29] Nishitoh H (2012). CHOP is a multifunctional transcription factor in the ER stress response. J. Biochem..

[30] Krajewski M, Ozdowy P, D’Silva L, Rothweiler U, Holak TA (2005). NMR indicates that the small molecule RITA does not block p53-MDM2 binding in vitro. Nat. Med..

[31] Cong L (2013). Multiplex genome engineering using CRISPR/Cas systems. Science.

[32] Gandin V (2014). Polysome fractionation and analysis of mammalian translatomes on a genome-wide scale video link. J. Vis. Exp..

[33] Zaccara S (2014). p53-directed translational control can shape and expand the universe of p53 target genes. Cell Death Differ..

[34] Lindqvist LM (2012). Translation inhibitors induce cell death by multiple mechanisms and Mcl-1 reduction is only a minor contributor. Cell Death Dis..

[35] Shi Y (2014). ROS-dependent activation of JNK converts p53 into an efficient inhibitor of oncogenes leading to robust apoptosis. Cell Death Differ..

[36] Shenton D (2006). Global translational responses to oxidative stress impact upon multiple levels of protein synthesis. J. Biol. Chem..

[37] Saxton RA, Sabatini DM (2017). mTOR signaling in growth, metabolism, and disease. Cell.

[38] Roux PP, Topisirovic I (2018). Signaling pathways involved in the regulation of mRNA translation. Mol. Cell. Biol..

[39] Thoreen CC (2009). An ATP-competitive mammalian target of rapamycin inhibitor reveals rapamycin-resistant functions of mTORC1. J. Biol. Chem..

[40] Khoury MP, Bourdon J-C (2011). p53 isoforms: an intracellular microprocessor?. Genes Cancer.

[41] Aoubala M (2011). p53 directly transactivates Δ133p53α, regulating cell fate outcome in response to DNA damage. Cell Death Differ..

[42] Candeias MM, Hagiwara M, Matsuda M (2016). Cancer‐specific mutations in p53 induce the translation of Δ160p53 promoting tumorigenesis. EMBO Rep..

[43] Sidrauski, C., Mcgeachy, A. M., Ingolia, N. T. & Walter, P. The small molecule ISRIB reverses the effects of eIF2 α phosphorylation on translation and stress granule assembly. 1–16. 10.7554/eLife.05033. (2015). 10.7554/eLife.05033 PMC4341466 25719440

[44] Zyryanova AF (2018). Binding of ISRIB reveals a regulatory site in the nucleotide exchange factor eIF2B. Science.

[45] Donnelly N, Gorman AM, Gupta S, Samali A (2013). The eIF2α kinases: their structures and functions. Cell. Mol. Life Sci..

[46] Lin W-C (2012). Endoplasmic reticulum stress stimulates p53 expression through NF-κB activation. PLoS ONE.

[47] Qu L, Koromilas AE (2004). Control of tumor suppressor p53 function by endoplasmic reticulum stress. Cell Cycle.

[48] Axten JM (2012). Discovery of 7-methyl-5-(1-{[3-(trifluoromethyl)phenyl]acetyl}-2,3-dihydro-1 H -indol-5-yl)-7 H -pyrrolo[2,3- d]pyrimidin-4-amine (GSK2606414), a potent and selective first-in-class inhibitor of protein kinase R (PKR)-like endoplasmic reticulum kinase (PERK). J. Med. Chem.

[49] Boyce M (2005). A selective inhibitor of eIF2alpha dephosphorylation protects cells from ER stress. Science.

[50] Rajesh K (2013). eIF2α phosphorylation bypasses premature senescence caused by oxidative stress and pro-oxidant antitumor therapies. Aging.

[51] Issaeva N (2004). Small molecule RITA binds to p53, blocks p53–HDM-2 interaction and activates p53 function in tumors. Nat. Med..

[52] Yoshikawa N (2016). PRIMA-1MET induces apoptosis through accumulation of intracellular reactive oxygen species irrespective of p53 status and chemo-sensitivity in epithelial ovarian cancer cells. Oncol. Rep..

[53] Ahmed, A., Yang, J., Maya-Mendoza, A., Jackson, D. A. & Ashcroft, M. Pharmacological activation of a novel p53-dependent S-phase checkpoint involving CHK-1. *Cell Death Dis.* **2**, e160 (2011). 10.1038/cddis.2011.42 PMC3122121 21593792

[54] Kondrashov AV, Spriggs KA, Bushell M, Willis AE (2009). Co-ordinated regulation of translation following DNA damage. Cell Cycle.

[55] Zhu H (2015). RITA enhances irradiation-induced apoptosis in p53-defective cervical cancer cells via upregulation of IRE1α/XBP1 signaling. Oncol. Rep..

[56] Lin JH, Li H, Zhang Y, Ron D, Walter P (2009). Divergent effects of PERK and IRE1 signaling on cell viability. PLoS ONE.

[57] Lin JH (2007). IRE1 signaling affects cell fate during the unfolded protein response. Science.

