# Supplementary figures and images for: RITA requires eIF2α-dependent modulation of mRNA translation for its anti-cancer activity
Source: Cell Death Dis. 2019 Nov 7;10(11):845. doi: 10.1038/s41419-019-2074-3 (PMC6838152; doi:10.1038/s41419-019-2074-3)

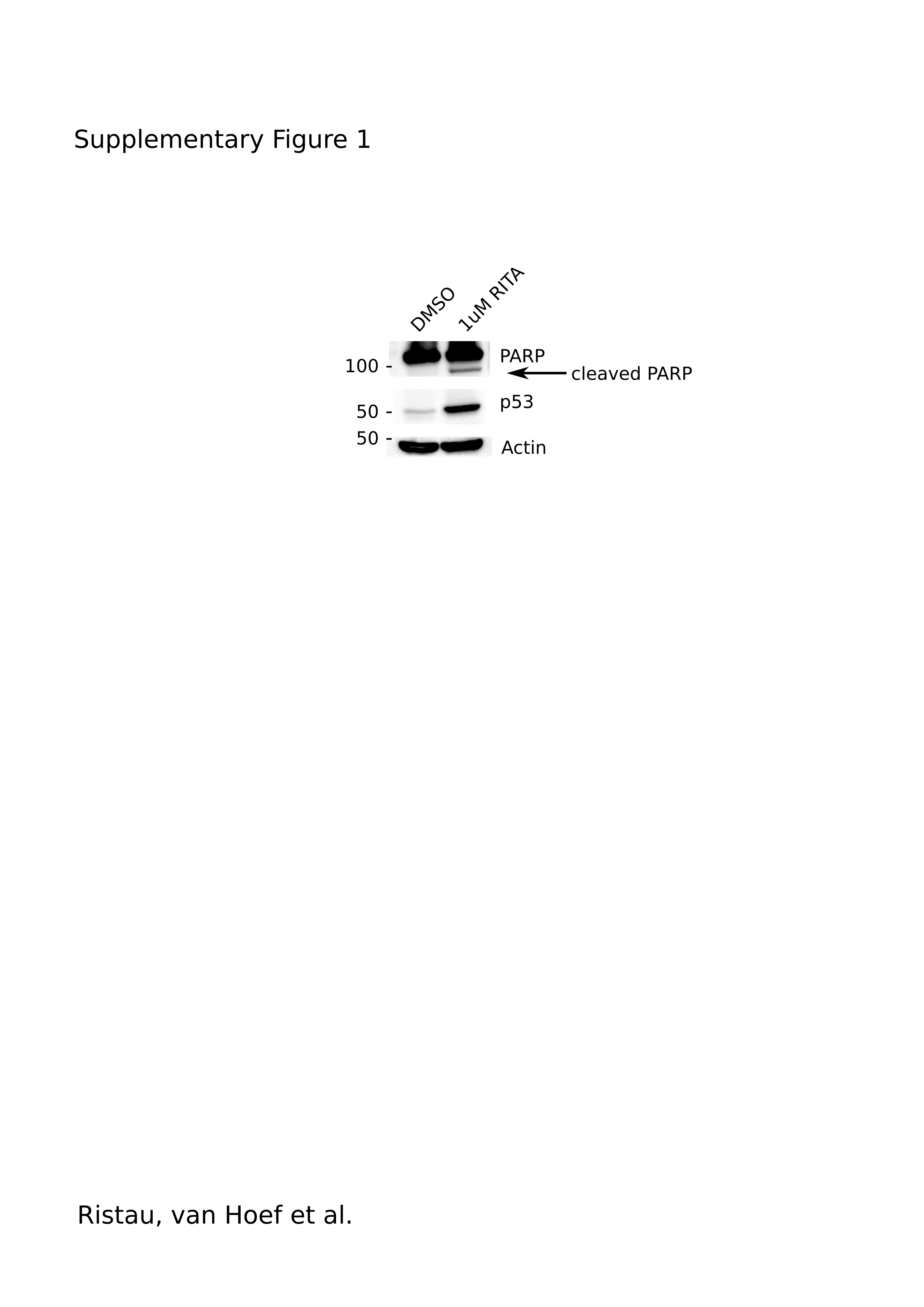

Supplement: Supplementary file 1 — Supplemental Figure 1 [file 41419_2019_2074_MOESM1_ESM.png]

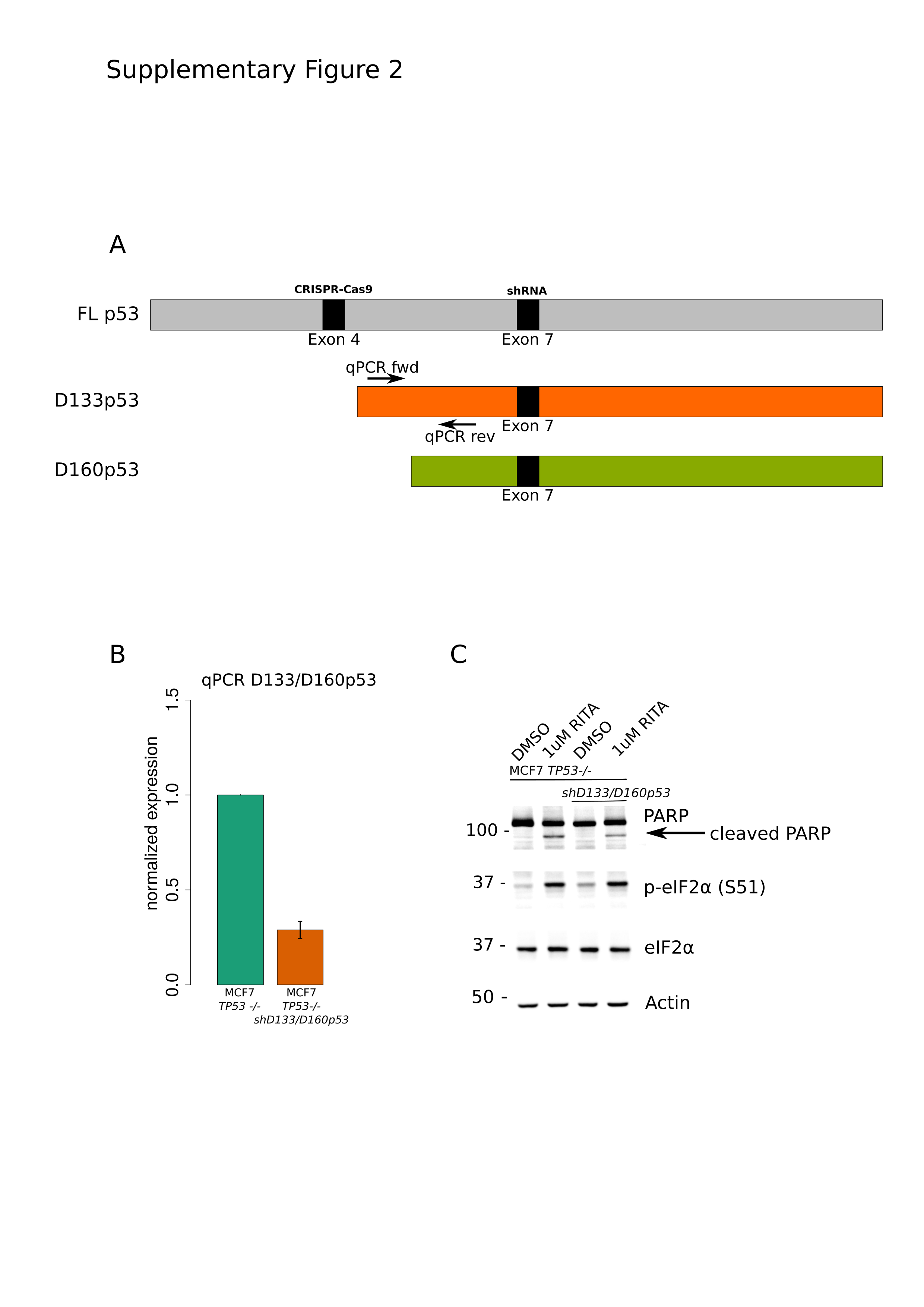

Supplement: Supplementary file 2 — Supplemental Figure 2 [file 41419_2019_2074_MOESM2_ESM.png]
