# Supplementary material for: RITA requires eIF2α-dependent modulation of mRNA translation for its anti-cancer activity
Source: Cell Death Dis. 2019 Nov 7;10(11):845. doi: 10.1038/s41419-019-2074-3 (PMC6838152; doi:10.1038/s41419-019-2074-3)
Supplement: Supplementary file 3 — Supplemental Figure Legends no marked changes [file 41419_2019_2074_MOESM3_ESM.docx]

**SUPPLEMENTARY FIGURE LEGENDS**

**Figure S1. RITA induces p53 and cleavage of PARP.**

Western blot analysis using whole cell extracts from MCF7 WT cells treated with vehicle (DMSO) or 1 µM RITA for 8 h.

**Figure S2. RITA induces PARP cleavage and eIF2α-phosphorylation independently of D133/D160p53.**

A) Comparison between full-length p53 (FL p53) and p53 isoforms (D133p53, D160p53) indicating exons targeted by CRISPR/Cas9 and shRNA. Annealing positions for qPCR-primers are indicated by arrows. B) Expression of D133p53 mRNA (which encodes both D133p53 and D160p53 protein isoforms) analyzed by qPCR (mean +/- SD from a technical triplicate). C) Western blot analysis using whole cell lysates from

control (DMSO) MCF7 TP53-/- and MCF7 TP53-/- shD133/D160p53 cells treated with 1 uM RITA for 16 h.
