# Supplementary material for: RITA requires eIF2α-dependent modulation of mRNA translation for its anti-cancer activity
Source: Cell Death Dis. 2019 Nov 7;10(11):845. doi: 10.1038/s41419-019-2074-3 (PMC6838152; doi:10.1038/s41419-019-2074-3)
Supplement: Supplementary file 4 — Author contribution file [file 41419_2019_2074_MOESM4_ESM.pdf]

DECLARATION OF CONTRIBUTIONS TO ARTICLE **ADMC**

Manuscript Number:	Journal Name:	
CDDIS-19-1524R	*Cell Death & Disease*	(the 'Journal')

Proposed Title of the Contribution:

RITA requires eIF2-dependent modulation of mRNA translation for its anti-cancer activity (the 'Contribution)

Author(s):

Johannes Ristau, Vincent van Hoef, Sylvain Peuget, Jiawei Zhu, Bo-Jhih Guan ,Shuo Liang, Maria Hatzoglou, Ivan Topisirovic, Galina Selivanova, Ola Larsson (the 'Authors')

For all *CDDis* articles, each person named as an author in the published version must be able to show he or she has contributed substantially to the article.

Authorship credit should be based on 1) substantial contributions to conception and design, acquisition of data, or analysis and interpretation of data; 2) drafting the article or revising it critically for important intellectual content; and 3) final approval of the version to be published. Authors should meet conditions 1, 2 and 3.

Any person who cannot be shown to have made a substantial contribution to the article cannot be listed as an author in the final version. The name of any person who is deemed to have made a minor contribution can, however, appear in the Acknowledgments section of the article.

Please complete the table below to indicate the contributions of all named authors to the manuscript.

Author Full Name:	Specification of Contribution to the Manuscript:
Johannes Ristau	Performed and designed experiments, interpreted the data and wrote the manuscript.
Vincent van Hoef	Performed and designed experiments, interpreted the data and wrote the manuscript.
Sylvain Peuget	Performed and designed experiments and interpreted the data.
Jiawei Zhu	Performed experiments
Bo-Jhih Guan	Performed experiments
Shuo Liang	Performed experiments
Maria Hatzoglou	Helped substantially to interprete the data.
Ivan Topisirovic	Helped substantially to interprete the data and provided cell lines.
Galina Selivanova	Designed the study and heleped substantially to interprete experiments, aquired funding.
Ola Larsson	Designed the study and helped substantially to interprete experiments, wrote and edited the manuscript, aquired funding.

Please complete the table below to indicate the contributions of all named authors to the figures.

Figure 1:

V.v.H. and J.R. performed experiments, V.v.H., J.R., G.S. and O.L. interpreted the data. J.R and V.v.H. assembled figures.

Figure 2:

V.v.H. and J.R. performed experiments, V.v.H., J.R., G.S. and O.L. interpreted the data. J.R and V.v.H. assembled figures.

Figure 3:

V.v.H. and J.R. performed experiments, V.v.H., J.R., G.S. and O.L. interpreted the data. J.R and V.v.H. assembled figures.

Figure 4:

V.v.H., S.P. and J.R. performed experiments, V.v.H., J.R., S.P., G.S. and O.L. interpreted the data. J.R., V.v.H. and S.P. assembled figures.

Figure 5:

V.v.H. and J.R. performed experiments, V.v.H., J.R., G.S. and O.L. interpreted the data. J.R and V.v.H. assembled figures.

Figure 6:

J.R. performed experiments, J.R., G.S. and O.L. interpreted the data. J.R assembled figures.

Signed for and on behalf of the Author(s):	Print Name:	Date:
	Ola Larsson	30:th September 2019
